# A Rare Presentation of NEC and High Grade IVH in a Near-Term, Normal Weight Baby With Positive Thrombophilia Profile: Case Report

**DOI:** 10.1155/crpe/8889033

**Published:** 2025-05-02

**Authors:** Maria Amr, Raja Imad Abu Iram, Ehab Mohammad Abuawwad, Iyad Zuhair Jabari, Areen H. Qunaibi, Walaa Altamimi, Ahmad Abu Sharkh

**Affiliations:** ^1^College of Medicine and Health Sciences, An-Najah National University, Nablus, State of Palestine; ^2^College of Medicine and Health Sciences, Palestine Polytechnic University, Hebron, State of Palestine; ^3^Department of Pediatrics and Neonates, Palestinian Red Crescent Hospital, Hebron, State of Palestine

**Keywords:** case report, intraventricular hemorrhage (IVH), near-term, necrotizing enterocolitis (NEC), thrombophilia

## Abstract

Necrotizing enterocolitis (NEC) is a serious condition characterized by severe ischemic inflammation of the bowel with invasion of gas-forming organisms into the bowel wall. Intraventricular hemorrhage (IVH) is another serious condition characterized by bleeding into the ventricles from the friable germinal matrix in premature infants. Both typically occur in the preterm and low–birth weight neonates. In this report, we present a 36+-week gestation and normal weight newborn with no risk factors developed both NEC and high-grade IVH. Upon investigation, he was found to have a positive thrombophilia profile.

## 1. Introduction

Necrotizing enterocolitis (NEC) and intraventricular hemorrhage (IVH) are common serious conditions that primarily affect gastrointestinal and neurological systems of preterm and low–birth weight infants requiring neonatal intensive care due to the high risk of morbidity and mortality as well as potential long-term complications [1]. It is rare for normal weight term and near-term neonates to present with either and even more rare when they are combined [2]. With major risk factors ruled out, it seems important to explore the role of coagulation abnormalities in such an infrequent combination. In this current study, we present a case of a late preterm infant with both Grade IV IVH and NEC on the second day of life. Further investigation revealed a positive thrombophilia profile, suggesting a potential link between thrombophilia on one hand and NEC and IVH on the other hand.

## 2. Case Presentation

This male patient is a newborn baby to a nonconsanguineous Palestinian couple; he was delivered to a healthy G2P2A0 mother by a normal spontaneous vaginal delivery (NSVD) at 36 + 2 weeks of gestation in a good general health with an Apgar score of 8 and 9 at 1 and 5 min, respectively. Birth weight was 2670 g, head circumference of 33 cm, and length of 50 cm. Vitamin K and Hepatitis B vaccine were given at birth.

Shortly after birth, he developed tachypnea, grunting, subcostal retractions with borderline oxygen saturation (SpO2) of 93%, for which he was admitted to the NICU as a case of transient tachypnea of newborn (TTN). He passed urine and meconium within the first 24 h after birth.

On the second day of life, on evaluation of the respiratory system, he started to have episodes of apnea with compensated metabolic acidosis on arterial blood gases (ABGs) as shown on ABGs (pH: 7.35, HCO_3_: 18 mmol/L, PCO_2_: 32 mmHg, and BE: −5.2), for which he was intubated for 12 h, then he was extubated to a noninvasive positive pressure ventilation (NIPPV) for 2 h, and then he remained stable on room temperature with acceptable blood gases. Chest X-ray shows signs of TTN.

**Figure 1:**
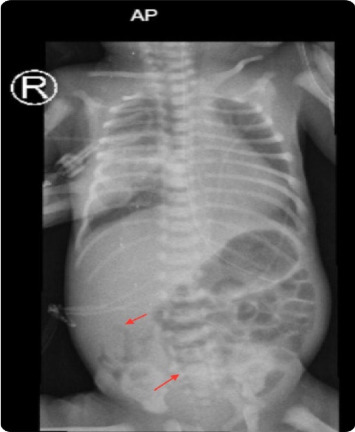
Supine abdominal X-ray showing air within bowel walls (red arrows) (pneumatosis intestinalis).

On gastrointestinal evaluation, he was being fed well by orogastric tube (OGT)—due to respiratory distress—until the age of 2 days when he developed abdominal distension with yellowish residue for which abdominal X-ray was done and showed air in the bowel wall (pneumatosis intestinalis), as shown in Figure 1, so he was kept nil per os (NPO) on total parenteral nutrition (TPN) with appropriate antibiotics for 10 days as a case of NEC Stage 2.

**Figure 2:**
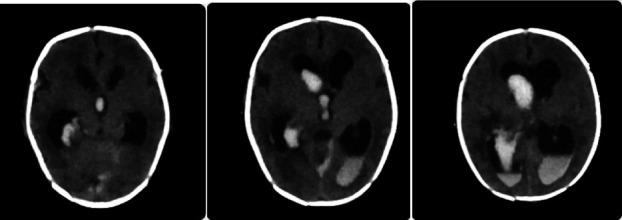
A brain CT scan showing IVH involving the subadjacent parenchyma consistent with Grade IV IVH with mild dilatation of the ventricular system.

On the central nervous system (CNS) evaluation on the second day after birth, the patient became hypoactive with mild spasticity of his four limbs, so transfontanel brain ultrasound (U/S) and lumbar puncture (LP) were done to rule out brain malformations, IVH, and infection. U/S was suggestive of IVH Grade 3 with normal brain parenchymal tissue. However, due to the low accuracy of our ultrasound machine and the current unavailability of an MRI, it was decided to perform brain CT scan for further evaluation. It was done and showed IVH Grade IV, as shown in Figure 2. Cerebrospinal fluid (CSF) analysis showed WBC 4 mg/dL “neutrophils 0%, lymphocytes 100%,” total cells 120, protein 388 mg/dL, and glucose 13 mg/dL, and CSF culture showed no growth.

The patient was hemodynamically stable with normal complete blood count (CBC) at birth. On Day 3 of life, he became hypotensive, his CBC at that time showed a drop in hemoglobin (Hgb) with low hematocrit and leukopenia (results detailed in Table 1), so he was given intravenous (IV) fluid and one packed red blood cells (PRBCS). C-reactive protein (CRP) and blood culture were taken to rule out sepsis, and empirical antibiotics (piperacillin-tazobactam and amikacin) were started, blood culture came back negative, and CRP was 10. After that, on Day 5 of life, his CRP was raised from 10 to 60 with neutropenia and new cultures were taken and antibiotics upgraded to meropenem and amikacin, and when all cultures came back negative, antibiotics were continued for total of 10 days for management of NEC.

Neonatal jaundice was developed at the age of 4 days due to ABO incompatibility (mother's blood group is O+, and baby's blood group is A+) with positive direct Coombs test (DCT) so he was appropriately managed with intensive phototherapy. Bilirubin remained below the level for exchange transfusion.

**Figure 3:**
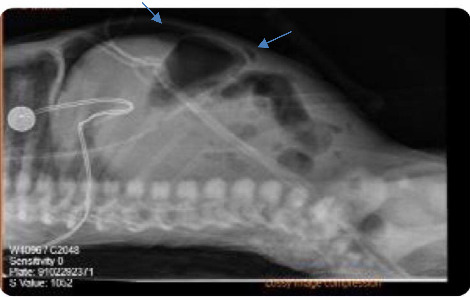
Supine lateral abdominal X-ray showing free intraperitoneal air (blue arrows) consistent with bowel wall perforation.

At the age of 6 days, he developed abdominal distension for which abdominal X-ray was repeated and showed evidence of bowel perforation, as shown in Figure 3, so urgent laparotomy was done, necrotic bowel was grossly visualized, and resection of 10 cm from the terminal ilium with primary anastomosis was performed. On postoperative follow-up, CBC showed Hgb of 10, so he was given a PRBCS, and he was kept NPO with abdominal drain and on TPN.

On follow-up at the age of 7 days, he was noticed to have inappropriately increasing head circumference (37.5 cm), so transfontanel U/S was done and showed ventriculomegaly consistent with post-IVH hydrocephaly, and regular brain ultrasound was done and showed progressive hydrocephalus for which neurosurgeon was consulted, and VP shunt was inserted.

Due to this unusual presentation of IVH Grade 4 and NEC with perforation, and after exclusion of all other possible causes that can lead to these conditions in this atypical patient, coagulation profile and thrombophilia panel were done, and the results were positive (details are shown in Tables 2 and 3).

## 3. Discussion

This case demonstrates a challenging combination of medical and surgical scenarios in a near-term infant who was delivered by NSVD to a healthy mother with an uncomplicated antenatal history, including the early onset IVH and NEC and the progressive deterioration of the overall clinical status after the development of post-IVH hydrocephalus and perforated NEC. The eventual identification of a significant thrombophilia profile potentially provides answers to the questions that have been circling around this patient's clinical course.

NEC is a critical neonatal emergency characterized by inflammation of the bowel causing bacterial invasion and coagulative necrosis of the colon and intestine [3]; according to the literature, term infants tend to develop NEC earlier than preterm infants, with the mean onset being within the first week of life while extending up to 20 days in preterm infants [4]. The initial tachypnea in this patient and his metabolic acidosis that was followed by bilious vomiting, abdominal distension, and radiologic evidence on X-ray that showed air in the bowel walls were highly suggestive of NEC [5]. In this case, other known risk factors such as prenatal factors, including maternal hypertensive disorders, drug use, and infections as well as birth asphyxia, congenital hypothyroidism, congenital heart diseases, and formula feeding were all absent [6].

The patient was managed with bowel rest TPN and antibiotics which was initially appropriate. However, the rapid escalation of events toward bowel perforation—on the sixth day of life—that was immediately operated confirmed the diagnosis by the visualized and resected necrotic bowel but was raising the doubts about the mechanism in which it happened since pathophysiology of NEC in neonates at such age or older involves reduced mesenteric perfusion [7], and the perforation was attributed to the leukopenia as well as the elevated CRP and the need of mechanical ventilation [8].

The unexpected NEC at this age necessitated additional workup; coagulation profile was ordered and an echocardiogram was done for the assessment of any congenital heart diseases and/or cardiac emboli. CT angiography for mesenteric vessels was not done because it is not available at our hospital and needs referral to a center which has this facility, but the clinical status of the patient did not allow it due to occurrence of perforation being too early before the noninvasive laboratory test results (the coagulation profile) came back.

Among the theories postulated to understand the pathological process of NEC being one that links it to thrombophilia, it presumes that NEC is caused by thromboemboli that travels through the mesenteric arteries occluding the artery, resulting in intestinal ischemia [9]. Given the known association between the FVL mutation and the increased risk of both arterial and venous thromboembolic events (VTEs) in neonates and children [10], it is plausible that this genetic factor was involved in the pathogenesis of NEC in this neonate.

Thrombophilia has not been established as a direct risk factor for NEC; however, it has been scientifically proven to be a significant risk factor for intestinal failure (IF). Neonates with IF are at increased risk for VTEs compared to the general pediatric population. The development of VTE has been attributed to a combination of factors including disease-specific factors and more generalized mechanisms such as inherited or acquired thrombophilia [11, 12].

IVH is another common complication in premature infants and low–birth weights; however, this near-term patient had an initial assuring Apgar score (8 and 9 at 1 and 5 min) with a negative history of maternal infection, evident birth trauma, birth asphyxia, or thrombocytopenia. At Day 2 of life, when the patient became hypoactive and developed four limbs spasticity, brain ultrasound and confirmatory CT showed IVH Grade IV which occurs only in 3.8% of preterm infants less than 31 weeks and is strongly associated with pregnancy complications especially placental abruption and inflammatory syndromes that both did not occur [13]. The IVH was concerning in terms of its grade, early onset, the infant's gestational age, and the absence of a risk factors that could be correlated to this rare incident. CSF analysis showed findings consistent with IVH (elevated cells count [RBCs], elevated protein, low glucose, and normal WBC count), and the patient was covered with broad-spectrum antibiotics until cultures results came back negative showing no bacterial growth.

Petäjä et al. suggest that IVH is one of the conditions that could be provoked by thrombophilic coagulation especially Gln506-FV, which is positive in our case, affirming that such genetic mutations predispose neonates to vascular thrombotic events despite unavailable risk factors [14]. The explanations proposed to justify the source of bleeding in the few cases of IVH in neonates ≥ 36 weeks were mostly ischemia in the thalamus and venous thrombosis related to choroidal plexus hemorrhage rather than fragility of the premature germinal matrix capillaries which is the main cause of IVH in preterm [15, 16] which led to the suggestion that a neonate with a 36-plus-week presenting with IVH should be evaluated for cerebral sinovenous thrombosis (CST) [17]. A limitation to this case study is that computed tomography venography (CTV) and/or magnetic resonance venography (MRV), which are the most effective imaging techniques used to diagnose CST, were not performed [18].

This infant had a heterozygous change in Factor V Leiden which is the most common hereditary thrombophilic mutation in the Middle East and is known to increase the thrombosis risk by seven folds with primary and recurrent venous thromboembolism risk increased three to six folds [19–21].

The combination of the heterozygous MTHFR (Ala1298Cys) and homozygous MTRR (Ala66Gly) genetic mutations causes markedly reduced MTHFR enzyme activity with subsequently increased homocysteine levels which has an established correlation with arterial thrombosis as well as an increased overall risk for venous thrombosis [22].

## 4. Conclusion

Term and near-term neonates presenting with vascular events such as NEC and IVH with no risk factors such as asphyxia, septicemia, dehydration, indwelling central line, congenital heart diseases, and maternal diabetes should undergo thrombophilia testing to check for genetic mutations explaining the conditions and for future prophylactic reasons [23].

## Figures and Tables

**Table 1:** CBC results in first 5 days of life.

Blood count	1st day	2nd day	3rd day	4th day	5th day	Reference range
Hemoglobin	16.4	14.6	10.3	9.7	11.6	12–18
Hematocrit	49.2	43.8	30.9	29.1	34.8	37–51
WBCs	14.72	10.3	3.2	5.05	7.06	4.1–10.9

**Table 2:** Coagulation profile results.

Test name	Result (%)	Reference range (U/mL) (%)
Protein C	59	{28–124}
Protein S	76	{29–162}
Antithrombin	110	{39–97}

**Table 3:** Thrombophilia panel results.

Genetic factor	Result
MTHFR (Ala1298Cys)	Heterozygous
Factor V leiden (FV Arg506Gln)	Heterozygous
B-fibrinogen (Gly544Ala)	Heterozygous
MTRR (Ala66Gly)	Homozygous

## Data Availability

The data that support the findings of this study are available on request from the corresponding author. The data are not publicly available due to privacy or ethical restrictions.
